# Management of Fungal Rhinosinusitis: Experience From a Tertiary Care Centre in North India

**DOI:** 10.7759/cureus.23826

**Published:** 2022-04-04

**Authors:** Seema Monga, Junaid Nasim Malik, Arun Sharma, Deepti Agarwal, Ratna Priya, Khaja Naseeruddin

**Affiliations:** 1 Department of Otorhinolaryngology & Head and Neck Surgery (ENT-HNS), Hamdard Institute of Medical Sciences and Research, Hakeem Abdul Hameed Centenary Hospital, New Delhi, IND

**Keywords:** otorhinolaryngology, liposomal amphotericin, invasive fungal rhinosinusitis, endoscopic sinus surgery, aspergillus, allergic fungal rhinosinusitis

## Abstract

Introduction

Fungal rhinosinusitis (FRS) has increased over the past few decades due to the rampant use of antibiotics, steroids, immunosuppressive drugs, increased incidence of HIV and uncontrolled diabetes. The current study reviews the types, clinical presentation, microbiology, histopathology and outcomes related to FRS in a tertiary care center in North India.

Methods

We retrospectively reviewed the clinical and follow-up records of patients diagnosed with FRS over three years. The data reviewed included clinical workup, ophthalmological profile, comorbidities, immunological status, radiological investigations, intraoperative and histopathological findings, treatment and follow-up records. In addition, we performed a descriptive analysis of the reviewed data.

Results

The study consisted of 30 FRS patients (16 male, 14 female). In that, 77% of cases were of allergic FRS, while fungal ball, chronic invasive, chronic granulomatous and acute invasive FRS represented 3%, 10%, 3% and 7% cases, respectively. The most common presentation in non-invasive forms was nasal obstruction, nasal discharge, hyposmia and polyposis, while it was facial pain and headache in the invasive varieties. After appropriate medical and surgical management through endoscopic sinus surgery, the recurrence rate in non-invasive and invasive fungal sinusitis was 16.6% and 20.8%, respectively. There was nil mortality at a minimum of one year of follow-up.

Conclusion

The non-invasive forms of FRS are common and have a relatively mild course. Early medical and surgical intervention and management of the underlying comorbidities are the key factors in managing invasive FRS. Close follow-up after surgery is also necessary for the timely detection and management of recurrences.

## Introduction

Fungal rhinosinusitis (FRS) has been gradually increasing over the past few decades, owing to the rampant use of antibiotics and steroids [1]. It is characterized by inflammation of sinus mucosa due to fungal infection. Various species of fungi are responsible for the causation of fungal sinusitis, Aspergillus species being the most common [2]. FRS is broadly categorized as invasive and non-invasive [3]. Invasive FRS is usually seen in immunocompromised patients and includes acute invasive FRS (AIFRS), granulomatous invasive FRS (GIFRS), and chronic invasive FRS (CIFRS) [4-9]. Non-invasive FRS can be further divided into allergic FRS (AFRS) and fungal balls [3,10-13].

The usual treatment for FRS involves surgery and aggressive medical management with close follow-up [4-13]. The medical management includes anti-allergic medications, immunotherapy, and the addition of oral corticosteroids. Although medical management improves patient outcomes, more evidence regarding FRS management is still needed considering the risk of recurrence rates.

There has been limited evidence on the outcome-related characteristics of FRS in Indian populations. Literature suggests a high prevalence of AFRS among Indian populations [1]. However, the disease patterns and their outcomes can be affected by regional variations. Therefore, the current retrospective study reviews the types, clinical presentation, microbiology, histopathology and outcomes related characteristics of FRS in a tertiary care center in North India. The study findings can help understand the disease patterns and prognosis in the regional population.

## Materials and methods

We retrospectively reviewed the clinical and follow-up records of patients treated in the department of otorhinolaryngology and head and neck surgery of a tertiary care medical center over three years from January 2017 to December 2019. Only those patients with a diagnosis of FRS and a minimum of one-year follow-up were reviewed. The clinical records were reviewed for the demographic characteristics, clinical features, associated comorbidities, ophthalmological findings, immunological status, radiological investigations, intraoperative and histopathological findings, treatment, and follow-up data, including complications. The demographic parameters included number, gender, age, and mortality characteristics. The clinical features included signs and symptoms of the reviewed patients and their distribution among the invasive and non-invasive forms of FRS. The associated comorbidities included acute and chronic disorders requiring medical or surgical attention, such as diabetes mellitus (DM), hypertension, renal disorders, and any disorders affecting the patients' immunity. The ophthalmological findings were reviewed from the ophthalmic specialist consultation records. Such consultations have routinely been done in all patients with FRS in our institute. The special radiological investigations performed to know the extent of the disease, such as computed tomography (CT) and magnetic resonance imaging (MRI), were also noted. The intraoperative and histopathological findings as per the operative notes and biopsy reports were noted, and their contribution to the diagnosis formulation was assessed. The treatment and follow-up data were reviewed from the inpatient and outpatient files to review the medical and surgical management, recurrences, associated complications, and mortality. We performed a descriptive analysis of the reviewed data. The discrete variables were expressed as numbers, the categorical variables were expressed as percentages or proportions, and the continuous variables were expressed as means.

## Results

A total of 30 cases were reviewed. All belonged to North Indian states. The mean age was 41.2 years (17-62 years). The mean follow-up was 21.4 months. The clinical and demographic parameters are shown in Table 1.

**Table 1:** Clinical and demographic characteristics of the reviewed cases. AFRS: allergic fungal rhinosinusitis, AIFRS: acute invasive fungal rhinosinusitis, CIFRS: chronic invasive fungal rhinosinusitis, GIFRS: granulomatous invasive fungal rhinosinusitis

Parameters	Observations
Total number of cases	30
Mean age (years)	41.2
Male-female distribution	16 males and 14 females
Diagnosis based distribution, n (%):	
AFRS	23 (77%)
Fungal ball	1 (3%)
AIFRS	2 (7%)
CIFRS	3 (10%)
GIFRS	1 (3%)
Comorbidities and the number of associated FRS cases:	
Diabetes mellitus	4 (2 AIFRS, 2 CIFRS)
Hypertension	5 (2 AIFRS, 3 CIFRS)
Chronic kidney disease	2 (1 AIFRS, 1 CIFRS)
Hepatitis B	1 (1 AIFRS)

The clinical records revealed that all patients underwent a thorough clinical history and examination, after which diagnostic nasal endoscopy (DNE) was performed in all cases to collect swabs and biopsies to assess etiology. The distribution of clinical features among the invasive and non-invasive forms of FRS has been shown in Table 2. Appropriate radiological investigations, such as CT or MRI, were performed to confirm and categorize FRS and to see any impending complications like intraorbital extension, intracranial extension, the status of sinus mucosa, bone, cavernous sinus, and lamina papyracea. General blood investigations were carried out to assess general conditions and immunological status.

**Table 2:** Distribution of signs and symptoms in patients of fungal rhinosinusitis.

Clinical signs and symptoms	Patients with non-invasive fungal rhinosinusitis (n=24)	Patients with invasive fungal rhinosinusitis (n=6)
Nasal obstruction	23	3
Nasal discharge	23	2
Facial pain	12	6
Posterior nasal discharge	18	Not present
Headache	12	6
Hyposmia	23	Not present
Polyposis	23	Not present
Deviated nasal septum	8	2
Proptosis	3	2
Telecanthus	1	2
Fever	Not present	2
Abnormal vision	1	2
Facial paresthesia	Not present	1

Allergic FRS

Based on Bent and Kuhn criteria, 77% (n=23) cases were diagnosed AFRS [12]. Identification of fungus could not be accomplished in every case of AFRS. However, in potassium hydroxide staining, the presence of fungal hyphae was seen in 72% of AFRS cases. In the rest of the AFRS cases, the diagnoses were made based on clinicoradiological findings, raised Immunoglobulin E (which was raised in 89% of AFRS cases), histopathological findings or other minor criteria of Bent and Kuhn [12]. Among these patients, the most frequent comorbidities were allergic rhinitis 23 (100%) and bronchial asthma in eight (34.7%) cases. All of them had nasal obstruction, nasal discharge and hyposmia.

Additionally, 18 cases (78.2%) complained of postnasal drip. Nasal polyposis was also observed in all cases. CT paranasal sinuses showed heterogeneous serpiginous opacities, bony erosion, mucosal edema, and polyposis. All patients were kept on oral and topical nasal steroids preoperatively to decrease disease load and improve visualization during surgery. Functional endoscopic sinus surgery (FESS) was performed on all patients. The surgery aimed to have well-ventilated sinuses to decrease the recurrence risk and help in postoperative steroid spray application locally. Postoperatively, oral steroids were continued for two weeks in tapered doses along with two to six months of topical fluticasone spray to prevent recurrence of the disease. Eighteen out of 23 (78.2%) patients improved symptoms after surgery, five (21.7%) patients had disease recurrence and had to undergo revision surgeries. All patients were symptom-free by one year of follow-up.

Fungal ball

We encountered a single case of the fungal ball. Acute onset headache and diminution of vision were the presenting symptoms. DNE was suggestive of the deviated nasal septum to the left with a bulge seen in spheno-ethmoidal recess. Still, no mucosal discharge or polyp was seen in the nasal cavity. An MRI of paranasal sinuses revealed sphenoid sinus opacification and widening with optic canal dehiscence. The T1 images showed mild hyperintensity to muscle while the T2 image showed a complete lack of signal, characteristic of a fungal ball. Also, fungal hyphae were found on potassium hydroxide mount. The patient was administered high-dose prednisolone for three days preoperatively, considering the sudden visual loss. The patient underwent FESS with optic nerve decompression and had immediate recovery of visual acuity. No recurrence was observed at one year of follow-up.

Acute invasive FRS

Six patients were grouped under Invasive FRS, out of which two were of AIFRS. Both patients initially presented with sudden visual loss, with proptosis present in one. DNE revealed black crusts over the middle turbinate. Both patients were suffering from DM and chronic hypertension. CT findings included heterogeneous opacities, mucosal thickening, thinning lamina papyracea, and intraorbital extension. MRI with gadolinium enhancement showed a high signal on T2 weighted image with extraocular muscle thickening in one patient. Still, optic canals were disease-free in both cases. Potassium hydroxide mount showed broad aseptate hyphae, suggestive of mucormycosis. Endoscopic debridement was carried out in both patients. Histopathology was confirmative of mucormycosis with the invasion of hyphae in the mucosa and blood vessels. Intravenous amphotericin B was administered at a dose of 1 mg/kg up to a maximum of 5 g, with alternate day monitoring of renal function in one patient. The second patient had chronic kidney disease (CKD) and was administered liposomal amphotericin B at a dose of 4 mg/kg up to a total of 4 g with alternate day renal function monitoring.

Additionally, the patient was administered Posaconazole 200 mg twice daily for two months as he had residual orbital disease sparing the optic canal. Weekly endoscopic debridement was done for both the patients until no discharge or remnant of disease were seen on DNE after completion of systemic antifungal therapy. Both patients were disease-free at one year of follow-up.

Chronic invasive FRS

CIFRS was diagnosed in 13% (three) of our total cases. The most common presenting feature in this group of FRS was facial pain and headache. Proptosis was seen in one patient; however, no patient had any vision abnormality. All three patients had DM, and one among them was suffering from CKD and hepatitis B. CT revealed opacification of sinuses and heterogeneous opacities in all thee patients. Bone thinning was evident in two of them. Additionally, one of the patients had hard palate involvement. A definitive diagnosis of invasive sinusitis was concluded on histopathology and culture studies which revealed mucormycosis in two of them and Aspergillus in one.

Granulomatous invasive FRS

We had only one patient with GIFRS. He presented with headaches, facial pain, and left eye proptosis. DNE revealed edematous mucosa with yellowish crusts on the middle turbinate and pus in the middle meatus. CT suggested left-sided maxillary and ethmoid involvement with extension into the orbit. GIFRS with noncaseating granuloma was diagnosed on histopathological examination with Aspergillus identification in the microbiological study.

All chronic invasive types underwent surgical debridement of necrotic tissue endoscopically, and infrastructure maxillectomy was done in one patient. None of the patients developed any postoperative complications. All of them were administered amphotericin B (1 mg/kg up to a maximum of 5 g), and liposomal amphotericin B (4 mg/kg up to a total of 4 g) was administered in the patients with CKD with daily creatinine monitoring. After completing the systemic antifungal course, they were kept on twice a week follow-up. One of the patients developed recurrence at a three-month follow-up and underwent revision subtotal maxillectomy. She was kept on oral Voriconazole 200 mg for three months and did not develop any recurrence at one year of follow-up. All patients were asymptomatic at one year of follow-up. No mortality was seen in our series.

## Discussion

The current study findings suggest that AFRS is the major cause of FRS in the North Indian population. There was no marked gender predominance in our series. The comorbidities were associated with the invasive forms of the FRS, which could be potential risk factors as well. The clinical features suggested that nasal obstruction and discharge-related symptoms were more notable in non-invasive varieties of FRS, while ophthalmological complications were observed mainly in the invasive varieties. The clinical follow-up revealed favorable outcomes in general and with a risk of recurrences in mostly invasive varieties of FRS. Timely endoscopic debridement, medical management, and regular follow-up for recurrence management potentially contributed to the symptom-free follow-up and nil mortality in our series.

The mean age was approximately 41 years, and further review of records showed that most patients were in the 30-50 age group. The literature is heterogeneous concerning the age group affected with FRS. Milosević et al. found FRS more prevalent in male patients in adolescence and early adulthood [14]. In contrast, Satish et al. observed that most FRS patients belonged to the fifth decade [15]. Similarly, the gender predominance in literature has been heterogeneous. Chaganti et al. reported fungal sinusitis to be more common in females [16]. In some studies from Sudan and India, where AFRS was commonly seen in young adult males, it was postulated that these males commonly go to hot and dry climates and develop fungal sinusitis due to exposure and paranasal sinus mucosal injury in the field [17].

Noninvasive types of FRS are usually seen in immunologically competent people. AIFRS usually affects immunologically suppressed patients, such as those with DM, HIV, hematological malignancies, or those on immunosuppressive medications [18]. On the other hand, CIFRS and GIFRS can be found in either population [19]. A similar trend was observed in our findings, with invasive forms having associations with comorbidities. Like the previous studies, we also observed AFRS as the most prevalent FRS type [7,9,16]. On the other hand, few authors found fungal balls as common as AFRS [20,21]. The geographic heterogeneity may be due to diverse climatic conditions and variations in host factors like age and comorbidities.

In our study, most patients with non-invasive sinusitis presented with nasal obstruction, nasal discharge and hyposmia (95.8%), followed by postnasal drip (75%) (Table 2). In patients with invasive sinusitis, facial pain and headache were more common (100%). The findings are in line with the observations of previous studies [15,22]. In our series, 76.6% of patients had nasal polyps on examination. In the study by Waghray et al., 60% of patients had nasal polyposis [22]. However, in a study by Shah et al., 49% had nasal polyposis [23].

Our study had the limitation that the identification of fungal species could not be done in most cases due to the lack of infrastructure and financial restraint. In the literature, it is seen that Aspergillus species are the most common organism responsible for FRS. Depending on geographical location, it can either be Aspergillus fumigatus or Aspergillus flavus [24]. In our study, Rhizopus Oryza was the most common fungus isolated from samples of invasive fungal sinusitis. The diagnosis of 23 patients of AFRS in this study was based on Bent and Kuhn criteria, including immunoglobulin E mediated allergy to fungi, nasal polyposis, characteristic radiological findings, presence of eosinophilic mucin, and identification of fungus either by special stains or by culture [12]. Another limitation is the small sample size, which could be related to the cross-sectional nature of the study. Thus, any epidemiological conclusions cannot be made through the findings.

The standard treatment of AFRS is FESS to clear all polyps, eosinophilic mucin, fungal elements, re-establishing drainage pathways, and open the sinuses for better drug delivery [25]. Regular follow-up and medical management after surgery are important to prevent recurrence and facilitate normal mucosal function to resume. However, the exact medical management of perioperative conditions is debatable. In their systematic review, Gan et al. concluded that oral and topical nasal steroids should be given in post-surgical cases of AFRS and oral and topical antifungals, and immunotherapy may be used in refractory AFRS [26]. In our study, we performed FESS in all our AFRS patients and gave preoperative and postoperative oral and topical steroids, but we did not use oral antifungals or immunotherapy. Different studies have reported different recurrence rates of AFRS ranging from 25% to 80%, but the responsible factors for the recurrences are not well defined [27]. In the majority of the cases, insufficient disease clearance, poor follow-up, and inadequate postoperative treatment may be held responsible. In our study, the recurrence rate was 20.8% in AFRS patients.

The standard treatment of invasive FRS comprises management of underlying comorbidities, prompt surgical debridement, and systemic antifungal agents. Conventionally, radical surgical resection along with intravenous amphotericin B has been used. But recent studies favor the clearance of as much disease as possible and individually tailored surgical intervention as per the disease extent [18]. The duration of treatment with antifungals in the study by Gumaa et al. was up to 65 weeks [18]. In our study, early surgical debridement was done in all invasive sinusitis patients and management of underlying comorbidities. Immediate initiation of suitable antifungal agents was done in our study. The documented mortality rate of invasive FRS is 18% to 80% [28]. Fortunately, all of our patients were symptom-free after one year of follow-up. Early and effective management of underlying comorbidities could be favorable in our study, leading to zero mortality. However, a longer follow-up is required to analyze the recurrence and survival outcomes.

## Conclusions

Early surgical debridement, management of underlying comorbidities, and immediate initiation of a suitable antifungal agent are the key factors in managing invasive fungal sinusitis. In patients with AFRS, there is a potential role for preoperative and postoperative topical and nasal steroids. During FESS of AFRS patients, all polyps, eosinophilic mucin and fungal elements should be cleared. Furthermore, frequent follow-up and medical management after surgery in fungal sinusitis patients is important to prevent a recurrence. Further evidence in the form of large sample clinical and epidemiological studies would help in better understanding of FRS characteristics in the North Indian population.
